# Consumption of Goat Cheese Naturally Rich in Omega-3 and Conjugated Linoleic Acid Improves the Cardiovascular and Inflammatory Biomarkers of Overweight and Obese Subjects: A Randomized Controlled Trial

**DOI:** 10.3390/nu12051315

**Published:** 2020-05-05

**Authors:** Cristina Santurino, Bricia López-Plaza, Javier Fontecha, María V. Calvo, Laura M. Bermejo, David Gómez-Andrés, Carmen Gómez-Candela

**Affiliations:** 1 Nutrition Research Group, Hospital La Paz Institute for Health Research (IdiPAZ), 28046 Madrid, Spain; cristina.santurino@idipaz.es (C.S.); laura.bermejol@salud.madrid.org (L.M.B.); cgcandela@salud.madrid.org (C.G.-C.); 2 Food Lipid Biomarkers and Health Group, Institute of Food Science Research (CIAL, CSIC), Campus of Autonomous University of Madrid, 28049 Madrid, Spain; j.fontecha@csic.es (J.F.); mv.calvo@csic.es (M.V.C.); 3 Department of Anatomy, Histology and Neuroscience, School of Medicine, Autonomous University of Madrid, 28049 Madrid, Spain; dgandres10@hotmail.com; 4 Pediatric Neurology Unit, Hospital Universitari Vall d’Hebron, VHIR, 08035 Barcelona, Spain; 5 Dietetic and Clinical Nutrition Department, La Paz, University Hospital, 28046 Madrid, Spain

**Keywords:** n-3 PUFA, CLA, cheese, blood lipids, dairy fat

## Abstract

This study examines the value of a goat cheese naturally enriched in polyunsaturated fatty acids (PUFA) (n-3 PUFA and conjugated linolenic acid (CLA)) as means of improving cardiovascular and inflammatory health. Sixty-eight overweight and obese subjects (BMI ≥ 27 and <40 kg/m^2^), with at least two risk factors for cardiovascular disease (CVD) in a lipid panel blood tests, participated in a randomized, placebo-controlled, double-blind, parallel designed study. The subjects consumed for 12 weeks: (1) 60 g/d control goat cheese and (2) 60 g/d goat cheese naturally enriched in n-3 PUFA and CLA. Diet and physical activity were assessed. Anthropometric and dual-energy X-ray absorptiometry (DXA) tests were performed. Blood samples were collected at the beginning and at the end of the study period. Changes in health status, lifestyle and dietary habits, and daily compliance were recorded. The consumption of a PUFA-enriched goat cheese significantly increased plasma high-density lipoprotein (HDL)-cholesterol, as well as in apolipoprotein B, and it significantly decreased high-sensitivity C-reactive protein concentrations compared to the control goat cheese (*p* < 0.05). The significant improvement of the plasma lipid profile and inflammatory status of people with risk for CVD due to the consumption of PUFA-enriched cheese suggests a potential role of this dairy product as an alternative to develop high nutritional value food in a balanced diet comprising regular exercise.

## 1. Introduction

Cardiovascular disease (CVD) is the leading cause of death worldwide. The major risk factors are well-established and are mediated mainly by hypertension, dyslipidemia, and smoking, as well as others such as obesity, elevated cholesterol, poor diet, and physical activity. Due to the significant influence exerted by diet and lifestyle, the current nutritional recommendations like controlling the amount and quality and quality of the fats consumed in the diet and salt intake, as well as regular physical exercise, are key to the prevention and treatment of CVD [1].

Within this framework, the effect of polyunsaturated fatty acids (PUFA), such as α-linolenic acid (ALA) n-3, has been demonstrated through mechanisms involving anti-inflammatory, anti-arrhythmic, and anti-thrombotic properties, which reduce low-density lipoprotein cholesterol levels (LDL-C) and, to a lesser extent, high-density lipoprotein cholesterol levels (HDL-C) when they replace saturated fatty acids (SFA) [2]. Even though these beneficial effects are well known, in the last 100 years, the dietary ratio of n-6/n-3 PUFA in modern Western diets has dramatically increased to 15-17:1, which has been associated with an increase of many other illnesses including inflammatory diseases and cholesterolaemia [3]. In addition, some clinical trials in humans have indicated that conjugated linolenic acid (CLA) may have several beneficial effects for health, such as improving the blood lipid profile related to CVD and diabetes [4]. However, it is important to know the exact dose of CLA and the duration of the treatment to know the biological effects [4].

In recent decades, a wide variety of functional foods have been designed to reduce some of the factors that induce cardiovascular risks and to improve health [5]. Thus, one possible way to increase PUFA consumption is to enrich foods that are regularly consumed by the majority of the population such as dairy products. Even though full fat dairy products consumption has long been considered a risk factor for cardiovascular health, such products contribute to the mean daily intakes of energy (11%), protein (14%), fat (17%), calcium (48%), phosphorous (24%), and vitamin A (27%). Though further studies are needed, a recent meta-analysis has demonstrated that dairy product consumption is not associated with CVD [6]. Some studies have even proposed a distinction between dairy and other food sources of SFA based on their different effects on blood lipids [7] and the possible cardioprotective effect of eating fermented dairy products [8].

Modulating milk FA composition through the ruminant feeding, particularly with oilseeds rich in PUFA, has shown to be a valuable tool to improve milk nutritional value [9]. In particular, goat´s milk possesses some inherent properties and a great nutritional quality determined by its lipid composition, which makes it an attractive alternative to developing dairy products with a high added value, like cheese. In this respect, our group developed and characterized a goat cheese naturally enriched in CLA and omega-3 [9] to be further employed in a clinical trial on cardiovascular risk prevention in humans.

Thus, a randomized controlled trial was performed in order to assess the effect of the consumption of that PUFA-enriched cheese in modulating blood lipids (total cholesterol (TC), HDL-C, LDL-C, triglycerides (TAG), apolipoprotein A1 (ApoA1), apolipoprotein B (ApoB), and free fatty acids (FFA), as well as other cardiovascular risk factors, such as inflammatory markers, in overweight and obese subjects.

## 2. Materials and Methods

The present study was registered at http://clinicaltrials.gov under the number NCT02630602.

### 2.1. Subjects

**Figure 1:**
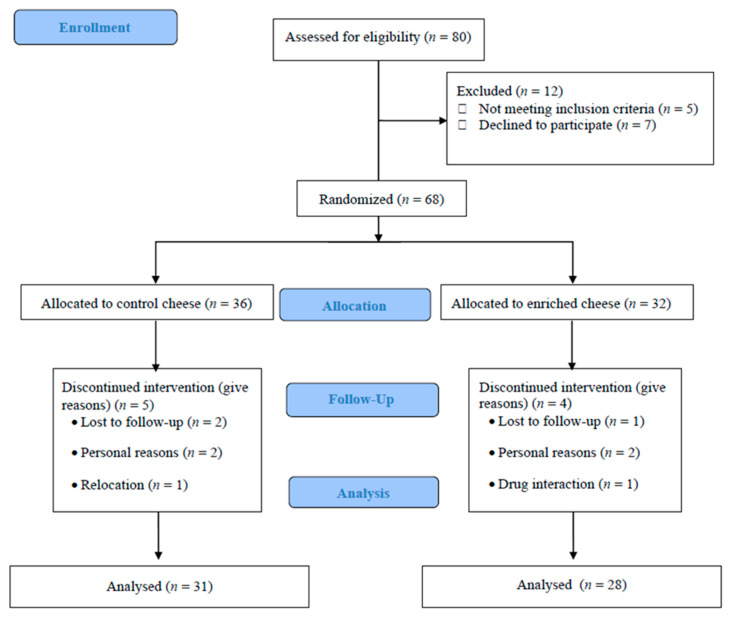
Flow chart describing the present trial.

For the present study, the Clinical Nutrition Department of La Paz University Hospital (HULP) in Madrid (Spain) recruited 68 overweight and obese subjects (52 women and 16 men) between January and March 2014. The inclusion criteria were: aged 18–65 years living in the region of Madrid, Spain; body mass index (BMI) ≥ 27 < 40 Kg/m^2^; to have a CVD risk score < 10% [10]; at least two atherogenic risk factors: TAG ≥ 150 mg/dL and <200 mg/dL, TC ≥ 200 mg/dL, HDL-C <40 mg/dL men or <50 mg/dL women, and/or LDL-C ≥130 mg/dL and <160 mg/dL, reflecting a risk for CVD [10]; having a suitable understanding of the clinical trial level; agreeing to voluntarily participate in the study; and signing the informed consent. Exclusion criteria were a diagnosis of diabetes mellitus, chronic degenerative diseases (e.g., liver or kidney), dyslipidemia, mental illness or diminished cognitive function, or the taking of antihypertension or lipid-lowering medication (e.g., statins, omega-3 supplements). Persons with lactose intolerance and dairy protein allergies were not enrolled. Pregnant or breastfeeding women were also excluded. The participants were individually allocated to one of the two study groups by randomization (Figure 1).

In addition, all groups followed the same balanced hypocaloric diet. All subjects gave their informed consent to take part in the study, which was approved by The Scientific Research and Ethics Committee of the Hospital Universitario La Paz (HULP 4092) and conformed to the ethical standards of the Declaration of Helsinki [11]; authorization for the disclosure of protected health information was obtained from all subjects before protocol-specific procedures. The participants were individually allocated to one of the two study groups, generated by a randomization procedure provided by the Biostatistics Unit of La Paz University Hospital. The allocation ratio of the study groups was 1:1.

### 2.2. Study Design

The controlled, randomized, double blind, parallel dietary intervention trial consisted of a 12-week investigation period (84 days). The control group (CG) received 60 g/day of a commercial goat cheese, and the experimental group (EG) received 60 g/day of the goat cheese that was naturally enriched with n-3 PUFA and CLA. Both control and enriched cheeses were produced as described by Santurino et al. (2017) [12]. Immediately after manufacture, the control and enriched cheeses were vacuum packed, refrigerated, and marked to maintain the conditions of blinding. Thus, neither the participants nor the researchers knew to which group the members belonged until the end of the study.

### 2.3. Dietetic, Physical Activity and Comorbidities’ Data

Balanced hypocaloric and personalized diets were individually prescribed for all participants. An energy restriction of approximately 400 kcal/day was prescribed depending on gender, age, BMI, nutritional habits, physical activity, comorbidities and previous dietary treatments. Dietary intake was recorded using a food frequency questionnaire and a “3-day food and drink record” validated for the Spanish population [13] for computing energy, fat and protein intake. Two weekdays and one weekend day were included in the dietary record to take any differences in nutrient intake during weekdays and weekends into account. This was achieved by guidance from our dietitian. Subjects attended the department to collect the test food and for follow-up every three weeks throughout the intervention period. A questionnaire was fulfilled to collect the current use of medications and supplements, and the presence of relevant previous diseases and a physical activity metabolic equivalent of task (MET) score was determined based on self-reported energy-consuming activities during work, at home, while travelling, and at leisure time based on “Global Recommendations on Physical Activity for Health” by the WHO.

### 2.4. Anthropometric Variables

Blood pressure and heart rate were measured three times at 5-min intervals on the right arm using a Welch automatic monitor (Allyn Spot Vital Signs 420 series, Amsterdam, The Netherlands) (accuracy ±5 mmHg). The measurements were taken with subjects sitting, and the means were calculated. Dual-energy X-ray absorptiometry (DXA) was used to measure the total fat mass (TFM (%)), bone mineral density (BMD (g/cm^2^)), android fat (AF (%)), gynoid (GF), and the lean mass (LM (%)), employing a GE Lunar Prodigy apparatus (GE Healthcare, Madison, WI, USA). Finally, anthropometric measurements as subject composition (TANITA BC-420MA, Biológica Tecnología Médica S.L. Barcelona, Spain), BMI, and waist and hip circumference were measured and recorded while adhering to international norms set out by the WHO.

### 2.5. Blood Collection

Blood samples were taken at baseline and at the end of the study period after a 12 h overnight fast at the Extraction Unit of the Hospital Universitario La Paz (Madrid, Spain). Samples were collected early in a 5 ml vacutainer tube with EDTA, and they were centrifuged at 4 °C over 7 min at 3500 rpm. Finally, samples were kept at −40 °C until analysis. A biochemical serum lipid profile (TC, HDL and LDL cholesterol, triglycerides, apolipoprotein A1, apolipoprotein B, and free fatty acids), and glucose determinations were performed by an enzymatic-spectrophotometric assay using an Olympus AU 5400 apparatus (Izasa, CA, USA). C-reactive protein (CRP) concentrations were determined using a BNII nephelometer (Siemens Healthcare Diagnostics GmbH, Eschborn, Germany). Tumor necrosis factor-α (TNF-α) and interleukin 6 (IL-6) were determined using a Luminex ®-100 (Luminex Corporation. Texas City, TX, USA) multianalyte profiling system with commercially available immunoassay panels. Total lipid peroxides in plasma were determined as an indicator of oxidative stress by using the thiobarbituric acid reactive substances (TBARS) method21. The results were expressed as µmol MDAeq/mL. Data were analyzed using the xPONENT v.3.1 software (Merck Millipore, Burlington, VT, USA) and were determined using specific protocols of La Paz University Hospital.

### 2.6. Compliance and Adverse Events

Compliance was measured at the end of each experimental period using a specific questionnaire, and a subject was considered compliant when he/she consumed the contents of ≥70% of the product. Adverse events were recorded during the experimental periods. An adverse event was defined as any unfavorable, unintended effect reported by a subject or observed by the investigator. All were recorded along with the symptoms involved (nausea, vomiting, diarrhea, halitosis, and/or constipation). No participants showed any signs of intolerance to the supplement of the study diets. Subjects were informed of their right to withdraw from the study at any time.

### 2.7. Statistical Analysis

The sample size of 30 subjects in each group was calculated to provide 90% power at a 5% level of significance by the power analysis (nQuery Advisor Release 2.0, Statistical Solutions, Boston, MA, USA) based on LDL-C as a target effect size. The primary outcomes of the study were the changes from baseline to week 12 in the TC, low density lipoprotein cholesterol, and high-density lipoprotein cholesterol. Changes in triglycerides, free fatty acids, lipoproteins apoA-1 and apoB, fasting glucose, fasting insulin, body mass index, waist circumference, and the percent of fat tissue and its distribution assessed by android-to-gynoid fat percent ratio, as well as the total visceral adipose tissue, inflammatory markers (CRP, IL-6, TNF-α, oxidized low-density lipoprotein (OxLDL), and fibrinogen), calcium, phosphate, vitamin D, ghrelin, and leptin were considered as secondary outcomes. Baseline features in the intervention and control group were compared by a t-test (continuous variables) or by a chi-squared test (categorical variables). Changes in the primary and secondary outcomes from baseline to week 12 were defined by the absolute difference of the value of a parameter in week 12 minus the value at baseline. The statistical analysis of not normally distributed parameters were assessed by a Mann–Whitney U non-parametric test. The 95% confidence intervals of the absolute difference of the mean changes between the intervention and control groups were calculated by adjusted bootstrap percentile method after a 1000-replication bootstrap. Statistical calculations were performed in R (R Core Team (2013), Vienna, Austria).

## 3. Results

### 3.1. Recruitment and Study Population

Eighty possible patients were screened for enrolment in this study, but only sixty-eight met the inclusion and exclusion criteria and were randomized. The participant flow diagram is shown in Figure 1. Nine participants did not finish the study due to personal reasons, refusal to participate further, or relocations. Thus, fifty-nine subjects finished the 12-wk intervention period (control group: 31 subjects; experimental group: 28 subjects); only their data were included in analysis.

### 3.2. Baseline Characteristics

The baseline characteristics of the fifty-nine subjects who completed the study were found to be comparable between the two groups are described in Table 1. The treatment compliance was high, and no differences were observed between groups (>85% of the scheduled doses consumed in the CG; >87% in the EG; *p* < 0.374) (Table 1).

### 3.3. Dietetic and Anthropometric Variables

In general, the diets followed by the volunteers showed a similar intake of macro- and micro-nutrients. No significant baseline differences in the basal diet were noted among the groups, except for the weekly rations of legumes (*p* = 0.025) and water (*p* = 0.012), which were higher in the EG compared to the CG. Instead, weekly rations of meat (*p* = 0.021) were higher in the CG. Regarding the low number of adverse events reported, no conclusion towards a relationship with a specific intervention could be drawn. There was no significant change in body weight in either treatment group, nor in the BMIs after the 12 weeks of study (*p* > 0.05) (Table 1). Additionally, there were no significant differences between treatments in all the parameters of the DXA analysis, which allowed us to obtain accurate values of the variation of body composition (*p* > 0.05) (Table 1). Regarding the waist circumference, no significant differences were found in the baseline GC and EG values (*p* > 0.05).

### 3.4. Blood Pressure and Biochemical Variables

On the other hand, at the end of the intervention period, both systolic and diastolic blood pressure remained within normal values for the general population (120/80 mmHg). Though systolic blood pressure decreased by −5.21 ± 21.35 mm Hg in the EG there were no significant differences among groups, possibly due to intragroup differences, nor were there any significant differences at baseline or after the intervention for 12 weeks (*p* > 0.05) (Table 1).

The subjects’ blood lipids and apolipoproteins concentrations before and after intervention are shown in Table 2. At the end of the study, the level of TC increased significantly in the EG in comparison to the CG (*p* > 0.05), despite no significant difference from baseline observed in either group. Even though randomization, there was an imbalance between both groups in baseline HDL-C concentration (*p* = 0.04), with lower baseline HDL-C levels in the EG. However, this result was corrected, and a significant increase of HDL-C in favor of the EG occurred at the end of the intervention. The plasma levels of ApoA1 and ApoB remained within the reference values for the study population throughout the intervention period (Table 2). At the end of the intervention, no changes in ApoA1 levels (related to HDL-C, the most abundant apolipoprotein in plasma, which contributed to a good cardiovascular health [14]) were detected in any group. On the contrary, the plasma concentration of ApoB increased in the EG by the end of the intervention period (Table 2).

Additionally, the increase of TC in the EG could have been related to the significant increase of the HDL content in this group. Conversely, the consumption of cheese in both intervention groups did not significantly affect LDL-C values, leading to a significant improvement in the LDL/HDL ratio, a good lipid indicator of atherogenic risk along with the TC/HDL-C ratio (Table 3).

### 3.5. Inflammation Variables

Systemic inflammation (TNF-α, IL-6, CRP, and others) is described in Table 4. At baseline, there was no difference between groups in any of these characteristics. After intervention, there was a significant decrease in CRP in the EG by 36%, taking into account the intragroup variation.

Plasma calcium and phosphorous remained within the ranges of normality described throughout the intervention period (Table 5). At baseline, there was no difference between groups in the plasma levels of vitamin D, but there were lower than the reference range for the study population (20–40 ng/mL) in both the CG and the EG, maybe linked to overweight and obesity status [15]. In contrast, at the end of the intervention period, vitamin D plasma levels were found within values considered in the reference range for the study population [16]. There was also no significant change in ghrelin and leptin after the intervention in either group.

## 4. Discussion

This study was designed to evaluate the combined effect of a enriched cheese with a balanced hypocaloric diet and physical activity in overweight and obese subjects on cardiovascular risk factors. This diet enriched with n-3 PUFA and CLA did not significantly modify the body composition of either group. The lack of significant differences is in agreement with recent clinical trials in which the consumption of cheese naturally enriched with PUFA did not significantly modify the body composition of both healthy volunteers and subjects with altered lipid profiles [4,17]. Though the difference was not significant between both groups (*p* > 0.05), the reduction of the waist circumference in both intervention groups could be related to the good efficacy of the nutritional intervention and the guidelines for physical activity carried out in both intervention groups, thus decreasing the metabolic risk in relation to waist circumference specified by the WHO (>88 cm in women and >102 cm in men) (Table 1) [18]. These results are in accordance with those obtained in a recent clinical trial in which cheese consumption did not significantly modify anthropometric parameters related to metabolic risk among the different study groups [19]. Lastly, the slight non-significant decrease in heart rate observed at the end of the study in both groups may have been a consequence of weight loss, thus improving an important cardiovascular risk (CVR) factor [20] (Table 1). These results are in accordance with those obtained in a recent clinical trial in which a similar intervention period of 12 weeks has been previously shown to induce significant weight loss [21].

The DXA analysis revealed a baseline value of TFM that exceeded the typical values in overweight people (BMI 25–30 kg/m^2^) in both intervention groups. Though no significant differences were obtained between treatments, probably due to the low-calorie diet received by all volunteers (*p* > 0.05), at the end of the intervention, both groups had a slightly decreased TFM, AF and GF (Table 1). Recent studies have shown that weight loss and/or muscle mass could lead to a loss of BMD, thus highlighting the importance of a good dietary strategy in the management of overweight and obesity and avoiding the loss of muscle mass by performing regular physical exercise [22]. Consequently, the results showed the good follow-up of the recommendations for the daily performance of physical activity by all volunteers.

Regarding the increase in TC (the sum of HDL-C and LDL-C) in the EG, TC provides limited information about cardiovascular risk, and it is not useful for diagnosing metabolic syndrome [23] because it cannot be associated with a circulating increase in atherogenic lipoprotein concentration [24]. However, the significant increase in HDL-C in the EG after the intervention period was in line with another clinical trial in which hypercholesterolemic volunteers consumed PUFA-enriched yogurt for 10 weeks [25]. Furthermore, de Goede et al. (2015) in a recent review, concluded that the cheese intake as compared to butter might have beneficial effects on certain plasma lipids that are directly related to the antiatherogenic properties of CLA. Though the differences in the LDL-C/HDL-C ratio between the CG and the EG were not statistically significant (*p* > 0.05), the results showed a slight decrease in the atherogenic risk only in the EG at the end of the intervention. These results were in line with a recent study that evaluated the effect of the consumption of a LC n-3-PUFA-enriched cheese on the lipid profile in hypercholesterolemic adults [26]. In the study, no significant differences were found between both groups on plasma TAG (Table 2). This approach is in line with a recent review and meta-analysis where de Goede et al. (2015) concluded that cheese consumption has no effect on TAG levels in humans, and this effect could also be dependent on the intervention time [27]. Furthermore, a recent large review of nine RCT suggests that CLA did not significantly affect TC, TAG, or LDL-C contents [28]. On the contrary, Carrero et al. (2007) [29] supplemented hyperlipidemic volunteers with a milk product containing EPA plus DHA, and they observed a significant reduction in TAG and TC after eight weeks. In terms of increasing the plasma ApoB concentration in the EG, previous studies have reported significant changes in plasma ApoB concentration or even increases when prescribing therapies with LC n-3-PUFA, underscoring the importance of treatment duration to attain consistent results [30]. However, ApoB plasma levels in both groups were in the reference range of Apo B levels in adults, and there were no significant changes in plasma LDL-C levels, so this increase in ApoB levels cannot be considered an increase of cardiovascular risk [31]. The ApoB/ApoA1 ratio reflects the balance between two processes: the transport of cholesterol to peripheral tissues and the reverse transport to the liver. Due to the results obtained for the ApoA1 and ApoB values in both intervention groups, significant differences between the CG and the EG in the ApoB/ApoA1 ratio between before and after the intervention were not observed, but this ratio slightly increased at the end of the clinical trial in both groups. 

Weight loss is associated with reduced levels of pro-inflammatory cytokines responsible for inflammation, such as TNF-α and IL-6 [32]. Though recent studies have shown an increase in TNF-α and IL-6 levels due to the state of inflammation related to overweight and obesity [15], the consumption of 60 g/day of cheese, following a balanced and hypocaloric diet, kept these levels stable at the end of the intervention period (Table 4). These results were in line with those reported by Dawczynski et al. (2013) [7], where the consumption of PUFA-enriched yogurt did not significantly modify the values of inflammation markers studied in overweight and obese volunteers. Similarly, in our study at the end of 12 weeks of supplementation, there was no significant effect in plasma OxLDL, fibrinogen, and FFA compared with the control group, probably due to the wide variability among the volunteers in each group. These results were in line with those obtained by Joseph et al. (2011) [5] in an eight-week crossover clinical trial in which dietary supplementation with CLA-enriched oil did not modify plasma OxLDL values in overweight and hyperlipidemic subjects. Additionally, the plasma CRP concentration increases its levels in response to generalized inflammation, as in the case of overweight and obese individuals [33]. At the end of the intervention, the plasma CRP concentration increased by 37% in the CG, whereas this value, taking into account the intragroup variation, significantly decreased in the EG by 36%. This significant decrease in the plasma CRP concentration only in the EG did not coincide with previous studies in which the consumption of enriched dairy products in FA n-3 did not significantly modify the plasma levels of pro-inflammatory cytokines [33]. The lack of significant changes in levels of CRP has been also attributed to the duration of the intervention. Additionally, the reduction in CRP levels could be directly related to weight loss, decreasing 0.13 mg/dL CRP per kg of weight lost [33]. The consumption of FA n-3 increases their concentration in blood, cells, and tissues, and it alters the physical properties of cell membranes and the function of membrane proteins. FA n-3 is incorporated into cell membranes in competition with n-6 FA and AA. Considering that the replacement of n-6 FA with n-3 FA in membranes of the immune active cells may induces leucocytes to produce pro-inflammatory processes and lead to the reduced formation of pro-inflammatory compounds, the significant changes of plasma CRP levels in overweight and obese subjects only in the EG could have been due to the synergistic effect among the anti-inflammatory effect of the consumption of dietary FA n-3 and CLA, weight loss, and the consumption of a balanced diet, together with regular physical activity.

Calcium and phosphorus interact in numerous processes of the organism. Blood calcium values considered normal for a studied population are usually between 8 and 10.5 mg/dL, as well as between 2.4 and 4.5 mg/dL for phosphorus. For the population under study, the normal blood calcium and phosphorus values ranged between 8 and 10.5 mg/dL and between 2.4 and 4.5 mg/dL, respectively. In regards to vitamin D (which regulates mineral homeostasis, protects the integrity of the skeleton, and modulates cell growth and differentiation in a wide variety of tissues [34]), although there were no significant differences between both groups, the baseline levels of vitamin D in both the GC and the EG were lower than those considered normal for the study population (20–40 ng/mL), and this may be linked to overweight and obesity status [35]. On the other hand, hormonal regulators of satiety, such as ghrelin and leptin, are also related to body weight. Though no significant changes were seen in terms of intervention time and treatment group at the end of the clinical trial, an intragroup analysis revealed a slight decrease in leptin levels in both the GC and the EG. These results were expected after the hypocaloric diet and the consequent weight loss in both groups, as well as changes in the blood lipid profile [36].

## 5. Conclusions

Overall, the consumption of 60 g/day of cheese (both control and enriched), within the context of a balanced hypocaloric diet and recommendations for physical activity, was effective for the reduction of body weight, BMI and waist circumference in both the CG and the EG. Additionally, the healthy habits carried out by all subjects resulted in a slight decrease in heart rate, as well as maintenance of the BMD, resulting in a decrease in CVR. 

On the other hand, the significant increase of HDL and the significant decrease in blood levels of CRP in the EG improved the plasma lipid profile and the inflammatory status, thus producing a decrease in the atherogenic risk. Therefore, the consumption of this PUFA n-3 and CLA naturally enriched goat cheese could have a potential role as a high nutritional value food to improve the state of health.

## Figures and Tables

**Table 1:** Baseline characteristics and anthropometric parameters of the study participants before and after the intervention.

Characteristic	Week 0		Week 12		Week 12–Week 0		
	CG	EG	CG	EG	CG	EG	*p*–Value
Age (years)	47.60 ± 9.40	48.50 ± 7.80	-	-	-	-	-
Men (n)	8	6	-	-	-	-	-
Women (n)	23	22	-	-	-	-	-
Weight (kg)	85.6 ± 11.30	86.80 ± 15.80	82.18 ± 11.77	83.13 ± 15.75	−3.41 ± 3.13	−3.66 ± 2.46	0.865
BMI (kg/m^2^)	31.05 ± 3.30	30.74 ± 4.20	30.47 ± 3.69	30.54 ± 4.09	−1.12 ± 0.20	−0.93 ± 0.17	0.756
Waist circ. (cm)	105.0 ± 10.50	99.55 ± 10.60	96.29 ± 12.79	97.03 ± 11.09	−5.74 ± 6.63	−5.87 ± 3.18	0.889
BMD (g/cm^2^)	1.17 ± 0.12	1.16 ± 0.13	1.17 ± 0.12	1.16 ± 0.14	−0.004 ± 0.01	−0.002 ± 0.14	0.767
Lean mass (%)	45.23 ± 7.77	46.15 ± 9.72	44.75 ± 8.00	45.72 ± 9.84	−0.49 ± 1.24	−0.43 ± 1.21	0.723
Android fat (%)	51.62 ± 7.19	50.72 ± 7.14	48.83 ± 9.29	48.92 ± 7.82	−2.80 ± 2.99	−1.80 ± 2.44	0.436
Gynoid fat (%)	47.24 ± 7.53	47.22 ± 8.24	45.64 ± 8.22	45.20 ± 8.14	−1.60 ± 1.99	−2.02 ± 1.90	0.356
Total fat mass (%)	46.94 ± 5.87	46.27 ± 6.29	44.55 ± 6.87	43.11 ± 6.67	−1.79 ± 1.80	−1.72 ± 1.68	0.645
Systolic BP (mm Hg)	110.3 ± 14.00	110.9 ± 13.10	110.7 ± 10.09	105.64 ± 22.05	0.45 ± 9.41	−5.21 ± 21.35	0.123
Diastolic BP (mm Hg)	77.50 ± 10.70	76.50 ± 9.50	76.90 ± 8.53	75.07 ± 8.38	−0.61 ± 7.99	−1.46 ± 7.79	0.385
HR (rate per minute)	75.30 ± 10.50	77.70 ± 10.50	68.10 ± 11.97	75.71 ± 13.70	−7.23 ± 9.39	−1.96 ± 10.97	0.259

Data are expressed as the means ± SDs. Abbreviations: BMI: body mass index; Waist circ.: waist circumference; BMD: bone mineral density; BP: blood pressure; and HR: heart rate.

**Table 2:** Blood lipids and apolipoproteins concentrations before and after intervention.

(mg/dL)	CG			EG			Week 12–Week 0			
	Week 0		Week 12	Week 0		Week 12	CG	EG	CI 95%	
**TC**	201.80 ± 40.41		197.16 ± 36.95	195.39 ± 37.38		201.46 ± 38.81	−2 (−90; 43)	6.5 (−24; 50)	10.72 (0.37; 22.74)	^#^
**HDL-C**	54.97 ± 16.47	*	53.97 ± 11.08	47.89 ± 8.00	*	52.00 ± 9.24	2 (−48; 12)	4.5 (−5; 16)	5.11 (1.96; 11.46)	^#^
**LDL-C**	128.97 ± 31.34		125.61 ± 29.29	127.32 ± 32.45		129.71 ± 31.59	3 (−11; 16)	2.5 (−9; 31)	1.39 (−2.15; 5.92)	
**TAG**	89.00 ± 42.24		87.81 ± 47.65	100.71 ± 34.33		99.50 ± 31.59	6 (−65; 75)	1 (−56; 53)	−0.02 (−12.8; 14.62)	
**ApoA1**	161.32 ± 26.62		153.94 ± 20.85	150.68 ± 16.30		152.14 ± 18.40	−1 (−28; 9.6)	0.3 (−9.8; 24)	2.74 (−0.3; 6.98)	
**ApoB**	102.42 ± 21.45		101.65 ± 22.06	102.86 ± 23.33		108.00 ± 26.20	−2* (−19; 27)	3.5* (−20; 34)	5.92 (0.81; 11.77)	
**Glucose**	93.06 ± 8.08		95.39 ± 8.49	90.82 ± 9.40		94.54 ± 7.54	2.32 (70.04; 94.06)	3.71 (67.05; 95.04)	0.05 (–0.1; 0.19)	

Data are expressed as the means ± SDs. Statistical analysis was assessed by Mann–Whitney U test. * Significant difference between groups at week 0 *p* < 0.05. # Significant difference between groups at the end of the intervention period (week 0–week 12) *p* < 0.05. Abbreviations: TC: total cholesterol; HDL-C high-density lipoprotein cholesterol; LDL-C low-density lipoprotein cholesterol; and TAG triglycerides.

**Table 3:** Values of total cholesterol/high-density lipoprotein (TC/HDL), low-density lipoprotein (LDL)/HDL and apolipoprotein B/apolipoprotein A1 (ApoB/ApoA1) ratios before and after the intervention.

Ratio	CG (*n* = 31)		EG (*n* = 28)	
	Week 0	Week 12	Week 0	Week 12
**TC/HDL-C**	3.67 ± 2.5	3.67 ± 3.3	4.06 ± 4.6	3.87 ± 4.6
**LDL-C/HDL-C**	1.71 ± 1.9	1.72 ± 2.6	1.91 ± 4.0	1.79 ± 3.4 *
**ApoB/ApoA1**	0.63 ± 0.8	0.66 ± 1.1	0.68 ± 1.43	0.71 ± 1.42

Data are presented as mean ± s.d. * Significant difference between groups before and after the intervention *p* < 0.05. Abbreviations: TC: total cholesterol; HDL-C high-density lipoprotein cholesterol; LDL-C low-density lipoprotein cholesterol; and TAG: triglycerides.

**Table 4:** Inflammatory biomarker concentration before and after the intervention.

	CG		EG		Week 12–Week 0		CI 95%
	Week 0	Week 12	Week 0	Week 12	CG	EG	
**CRP (mg/L)**	2.67 ± 4.36	2.98 ± 7.62	2.95 ± 6.06	1.02 ± 6.33	0.03 (−18.12; 32.1)	−0.76 (−12.34; 3.38)	–10.72 (0.37; 22.74) ^#^
**TNF-α (pg/mL)**	3.88 ± 1.45	4.47 ± 1.43	4.51 ± 1.57	4.85 ± 1.71	0.5 (−0.8; 18.3)	0.75 (−1.2; 2.5)	−0.46 (−246; 0.28)
**IL-6 (pg/mL)**	2.60 ± 2.37	3.70 ± 2.25	2.70 ± 1.53	3.65 ± 1.60	1.2 (−1.7; 3.2)	0.95 (−2; 5.2)	−0.12 (−0.82; 0.6)
**Fibrinogen (mg/dL)**	397 ± 102.82	413 ± 97.33	419 ± 124.14	346 ± 129.64	−13 (−333;425)	−17 (−202; 106)	−26.88 (−81.27; 22.96)
**OxLDL (ng/mL)**	72.71 ± 238.10	80.53 ± 245.14	135.09 ± 229.32	113.47 ± 218.01	0.01 (−164.2; 654.9)	−0.31 (−250.9; 90.4)	−13.3 (−87.2; 16.9)
**FFA (mM)**	0.28 ± 0.17	0.25 ± 0.08	0.28 ± 0.11	0.23 ± 0.10	−0.01 (−0.47; 0.21)	−0.06 (−0.41; 0.2)	−0.03 (−0.09; 0.04)

Data are expressed as the means ± SDs. Statistical analysis was assessed by Mann-Whitney U test. Significant difference between groups at week 0 *p* < 0.05. # Significant difference between groups at the end of the intervention period (week 0–week 12) *p* < 0.05. Abbreviations: CRP: high-sensitivity C reactive protein; TNF-α: tumor necrosis alpha factor; IL-6: interleukin 6; oxLDL: oxidized LDL; and FFA: free fatty acids.

**Table 5:** Mineral and hormone concentrations before and after the intervention.

	CG		EG		Week12–Week 0		CI 95%
	Week 0	Week 12	Week 0	Week 12	CG	EG	
**Vitamin D (ng/mL)**	14.26 ± 6.15	21.89 ± 6.94	13.95 ± 6.06	22.5 ± 6.33	7 (1; 23)	8 (0; 17)	0.39 (−2.2; 2.26)
**Calcium (mg/dL)**	9.16 ± 0.38	9.24 ± 0.30	9.16 ± 0.32	9.30 ± 0.35	0.1 (−0.6; 0.7)	0.15 (−0.3; 0.9)	0.05 (−0.1; 0.19)
**Phosphorous (mg/dL)**	3.35 ± 0.39	3.41 ± 0.52	3.46 ± 0.59	3.32 ± 0.47	0.1 (−0.8; 0.8)	−0.1 (−1.4; 0.8)	−0.19 (−0.41; 0.02)
**Ghrelin (pg/mL)**	9.9 ± 13.75	10 ± 14.42	8.45 ± 19.62	8.10 ± 20.69	0.6 (−35.7; 40.3)	0.05 (−41.8; 39.2)	−0.37 (−7.72; 6.78)
**Leptin (pg/mL)**	14.14 ± 10.74	11.01 ± 10.00	14.80 ± 10.80	11.15 ± 10.50	−1.8 (−23.03; 13.1)	−2.7 (−9.5; 2.5)	−0.12 (−0.82; 0.6)

Data are presented as mean ± SDs.

## References

[1] Di Nicolantonio J., Lucan S.C. (2016). The evidence of saturated fat and for sugar related to coronary heart disease. Prog. Cardiovasc. Dis..

[2] De Goede J., Geleijnse J., Ding E., Soedamah-Muthu S. (2015). Effect of cheese consumption on blood lipids: A systematic review and meta-analysis of randomized controlled trials. Nutr. Rev..

[3] Dias C., Wood L., Garg M. (2016). Effects of dietary saturated and n-6 polyunsaturated fatty acids on the incorporation of long-chain n-3 polyunsaturated fatty acids into blood lipids. Eur. J. Clin. Nutr..

[4] Klok M., Jakobsdottir S., Drent M. (2007). The role of leptin and ghrelin in the regulation of food intake and body weight in humans: A review. Obes. Rev..

[5] Joseph S., Jacques H., Plourde M., Mitchell P., McLeod R., Jones P. (2011). Conjugated linoleic acid supplementation for 8 weeks does not affect body composition, lipid profile, or safety biomarkers in overweight, hyperlipidemic men. J. Nutr..

[6] Fontecha J., Calvo M.V., Juarez M., Gil A., Martínez-Vizcaino V. (2019). Milk and dairy product consumption and cardiovascular diseases: An overview of systematic reviews and meta-analyses. Adv. Nutr..

[7] Dawczynski C., Massey K., Ness C., Kiehntopf M., Stepanow S., Platzer M., Grun M., Nicolau A., Jahreis G. (2013). Randomized placebo-controlled intervention with n-3 LC-PUFA-supplemented yoghurt: Effects on circulating eicosanoids and cardiovascular risk factors. Clin. Nutr..

[8] De Oliveira Otto M., Nettleton J., Lemaitre R.M., Steffen L., Kromhout D., Rich S., Tsay M.Y., Jacobs D.R., Mozaffarian D. (2013). Biomarkers of dairy fatty acids and risk of cardiovascular disease in the multi-ethnic study of atherosclerosis. J. Am. Heart Assoc..

[9] Dittrich M., Jahreis G., Bothor K., Drechsel C., Kiehntopf M., Blüher M. (2014). Benefits of foods supplemented with vegetable oils rich in α-linolenic, stearidonic or docosahexaenoic acid in hypertriglyceridemic subjects: A double-blind, randomized, controlled trail. Eur. J. Nutr..

[10] Houston D., Driver K., Bush A., Kritchevsky S. (2008). The association between cheese consumption and cardiovascular risk factors among adults. J. Hum. Nutr. Diet..

[11] World Medical Association (WMA) (2013). Declaration of Helsinki. Ethical principles for medical research involving human subjects. JAMA.

[12] Santurino C., Calvo M., Gómez-Candela C., Fontecha J. (2017). Characterization of naturally goat cheese enriched in conjugated linoleic acid and omega-3 fatty acids for human clinical trial in overweight and obese subjects. PharmaNutrition.

[13] Khandelwal S., Demonty I., Jeemon P., Lakshmy R., Mukherjee R., Gupta R., Snehi U., Niveditha D., Singh Y., van der Knaap H.C. (2009). Independent and interactive effects of plant sterols and fish oil n-3 long-chain polyunsaturated fatty acids on the plasma lipid profile of mildly hyperlipidaemic Indian adults. Br. J. Nutr..

[14] Intorre F., Foddai M., Azzini E., Martin B., Montel M., Catasta G. (2011). Differential effect of cheese fatty acid composition on blood lipid profile and redox status in normolipidemic volunteers: A pilot study. Int. J. Food Sci. Nutr..

[15] Stone N., Robinson J., Lichtenstein A., Bairey Merz C., Blum C., Eckel R. (2013). 2013 ACC/AHA guideline on the treatment of blood cholesterol to reduce atherosclerotic cardiovascular risk in adults. Circulation.

[16] Kim B., Lim H., Lee H., Lee H., Kang W., Kim E. (2016). The effects of conjugated linoleic acid (CLA) on metabolic syndrome patients: A systematic review and meta-analysis. J. Funct. Foods.

[17] Klop B., Proctor S., Mamo J., Botham K., Castro Cabezas M. (2012). Understanding postprandial inflammation and its relationship to lifestyle behaviour and metabolic diseases. Int. J. Vasc. Med..

[18] López Gómez J., Pérez Castrillón J., Romero Bobillo E., De Luis Román D. (2016). Efecto del tratamiento dietoterápico de la obesidad sobre el metabolismo óseo. Nutr. Hosp..

[19] Marrugat J., Solanas P., D’Agostino R., Sullivan L., Ordovas J., Cordón F., Ramos R., Sala J. (2003). Estimación del riesgo coronario en España mediante la ecuación de Framingham calibrada. Revista Esp. Cardiol..

[20] Navarro-Alarcón M., Cabrera-Vique C., Ruiz-López M., Olalla M., Artacho R., Giménez R., Quintana V., Bergillos T. (2011). Levels of Se, Zn, Mg and Ca in commercial goat and cow milk fermented products: Relationship with their chemical composition and probiotic starter culture. Food Chem..

[21] Intorre F., Venneria E., Finotti E., Foddai M., Toti E., Catasta G. (2012). Fatty acid content of serum lipid fractions and blood lipids in normolipidaemic volunteers fed two types of cheese having different fat compositions: A pilot study. Int. J. Food Sci. Nutr..

[22] Nestel P., Mellett N., Pally S., Wong G., Barlow C., Croft K., Mori T.A., Meikle P.K. (2013). Effects of low-fat or full-fat fermented and non-fermented dairy foods on selected cardiovascular biomarkers in overweight adults. Br. J. Nutr..

[23] Pannu P., Calton E., Soares M. (2016). Calcium and vitamin d in obesity and related chronic disease. Advances in Food and Nutrition Research.

[24] Pariza M. (2004). Perspective on the safety and effectiveness of conjugated linoleic acid. Am. J. Clin. Nutr..

[25] Nilsen R., Høstmark A., Haug A., Skeie S. (2015). Effect of a high intake of cheese on cholesterol and metabolic syndrome: Results of a randomized trial. Food Nutr. Res..

[26] Obregón O., Gestne A., Lares M., Castro J., Stulin I., Rivas K. (2010). Estatinas y factor de necrosis tumoral alfa. Revista Latinoam. Hipertens..

[27] Piepoli M., Hoes A., Agewall S., Albus C., Brotons C., Catapano A., Cooney M.T., Corrà U., Bernard Cosyns C., Deaton C. (2016). European guidelines on cardiovascular disease prevention in clinical practice (version 2016). Eur. Heart J..

[28] Pranger I., Muskiet F., Kema I., Singh-Povel C., Bakker S.K. (2019). Potential biomarkers for fat from dairy and fish and their association with cardiovascular risk factors: Cross-sectional data from the lifelines biobank and cohort study. Nutrients.

[29] Salas-Salvadó J., Rubio M., Barbany M., Moreno B. (2007). Consenso SEEDO 2007 para la evaluación del sobrepeso y la obesidad y el establecimiento de criterios de intervención terapéutica. Med. Clín..

[30] Sánchez F., Albo Castaño M., Casallo Blanco S., Vizuete Calero A., Matías Salces L. (2008). Importancia de las apoproteínas A1 y B como marcadores de riesgo cardiovascular. An. Med. Interna.

[31] Shaikh N., Yantha J., Shaikh S., Rowe W., Laidlaw M., Cockerline C., Ali A., Holub B., Jackowski G. (2014). Efficacy of a unique omega-3 formulation on the correction of nutritional deficiency and its effects on cardiovascular disease risk factors in a randomized controlled VASCAZEN® REVEAL Trial. Mol. Cell. Biochem..

[32] Stone N., Robinson J., Lichtenstein A., Goff D., Lloyd-Jones D., Smith S., Blum C., Schwartz J.S. (2014). Treatment of blood cholesterol to reduce atherosclerotic cardiovascular disease risk in adults: Synopsis of the 2013 americcollege of cardiology/American heart association cholesterol guideline. Ann. Int. Med..

[33] Aguillón G.J., Cruzat C.A., Cuenca M.J., Cuchacovich T.M. (2012). El polimorfismo genético del factor de necrosis tumoral alfa como factor de riesgo en patología. Revista Méd Chile.

[34] Warensjo E., Jansson J., Cederholm T., Boman K., Eliasson M., Hallmans G., Johansson I., Sjogren P. (2010). Biomarkers of milk fat and the risk of myocardial infarction in men and women: A prospective, matched case-control study. Am. J. Clin. Nut..

[35] Aymé S., Rath A., Bellet B. (2018). WHO International Classification of Diseases (ICD) Revision Process: Incorporating rare diseases into the classification scheme: State of art. Orphanet J. Rare Dis..

[36] Castro I., Monteiro V., Barroso L., Bertolami M. (2007). Effect of eicosapentaenoic/docosahexaenoic fatty acids and soluble fibers on blood lipids of individuals classified into different levels of lipidemia. Nutrition.

